# Electroreduction of Bi(III) Ions at a Cyclically Renewable Liquid Silver Amalgam Film Electrode in the Presence of Methionine †

**DOI:** 10.3390/molecules26133972

**Published:** 2021-06-29

**Authors:** Agnieszka Nosal-Wiercińska, Marlena Martyna, Valentin Mirčeski, Sławomira Skrzypek

**Affiliations:** 1 Department of Analytical Chemistry, Institute of Chemical Sciences, Faculty of Chemistry, Maria Curie-Sklodowska University, Maria Curie-Sklodowska Sq. 3, 20-031 Lublin, Poland; marlena.martyna.96@gmail.com; 2 Department of Inorganic and Analytical Chemistry, Faculty of Chemistry, University of Lodz, Tamka 12, 91-403 Lodz, Poland; valentinmirceski@yahoo.com (V.M.); slawomira.skrzypek@chemia.uni.lodz.pl (S.S.); 3 Faculty of Natural Sciences and Mathematics, Institute of Chemistry, Ss. Cyril and Methodius University, Arhimedova 5, P.O. Box 162, 1001 Skopje, North Macedonia

**Keywords:** cyclically renewable liquid silver amalgam film electrode (R–AgLAFE), electrochemistry, electroreduction of Bi(III), active complexes, catalytic activity

## Abstract

The catalytic influence of methionine (Mt) on the electroreduction of Bi(III) ions on the novel, cyclically renewable liquid silver amalgam film electrode (R–AgLAFE) in a non-complexing electrolyte solution was examined. The presence of methionine leads to a multistep reaction mechanism, where the transfer of the first electron is the rate limiting step, which is the subject of catalytic augmentation. The catalytic activity of methionine is a consequence of its ability to remove water molecules from the bismuth ion coordination sphere, as well as to form active complexes on the electrode surface, facilitating the electron transfer process.

## 1. Introduction

The knowledge on the influence of organic molecules on electrode mechanisms is important for understanding reaction pathways and for technological and pharmacological applications based on electrochemical processes. It is known that molecular structures containing sulphur or nitrogen atoms, which are able to form coordination bonds and/or adsorb weakly on the electrode surface within a broad potential interval, frequently catalyze electrode processes in accordance with the “*cap*–*pair*” rule [1].

The catalytic effect of metal cation reduction according to the “*cap*–*pair*” mechanism includes both chemical reactions and heterogeneous charge transfer processes of active complexes on the electrode surface. The surface formation of active complexes within the electrode double layer with the studied metal cations is a plausible mechanism for zinc(II) [2,3], cadmium [4], indium(III) [5] and bismuth(III) ion reduction [6,7], while the complexation of europium(III) ions is assumed to proceed in the bulk of the solution as well [8].

It should be mentioned that the metal cations in aqueous solution exhibit strong interactions with water molecules. The dehydration steps play a big role in the deposition reactions. In acidic non-complexing electrolyte solutions the ${\left[\mathrm{Bi}{\left(H_{2}O\right)}_{9}\right]}^{3+}$ ion has a very low rate of hydration water loss. Therefore, the resulting electrode process includes chemical steps leading to labilization of the ${\left[\mathrm{Bi}{\left(H_{2}O\right)}_{9}\right]}^{3+}\;$ hydration shell [9].

In a general context, it is known that the study of an electrode mechanism is important for reliable estimation of the electrode kinetics [1,10]. Nowadays, thanks to modern electrochemical techniques, the mechanistic aspects of an electrode reaction can be studied in detail. The application of the innovative cyclically renewable liquid silver amalgam film electrode (R–AgLAFE) [10,11] appears to be a good alternative for the mercury electrode owing to the possibility to work in a wide range of potentials, which enables accurate determination of the electrode kinetic parameters. Moreover, substitution of mercury with silver is in the line with the general green chemistry trend.

In the present study the kinetics and mechanism of Bi(III) electroreduction in the presence of methionine (Mt) have been studied using R–AgLAFE. It is worth noticing that methionine is one of the amino acids essential for human life, frequently found in food containing large proteins [12]. Methionine is involved in many important biochemical processes such as methionine cycle, trans-sulphuration pathway and polyamine biosynthesis [13]. In the course of the metabolic pathways, it undergoes many chemical transformations [13]. Considering its applications for medical purposes, it is important to study its interactions with metal cations, in particular its effect on the reduction of various substances [12]. According to a study at the Duke University School of Medicine in Durham, a diet low in methionine may have an impact on the treatment of various diseases, in particular cancer [12,14,15]. Importantly, it has been found that methionine is involved in the cellular processes affected by chemotherapy drugs [14,15]. Thus, analytical determination of methionine is important from a bioanalytical point of view, as well as for understanding important biochemical reaction pathways.

## 2. Results and Discussion

### 2.1. Adsorption Measurements

The study of the differential capacity of the double layer, the potential of zero charge or determination of the surface tension at the potential of zero charge are important for revealing adsorption phenomena on the electrode surface [16,17].

**Figure 1:**
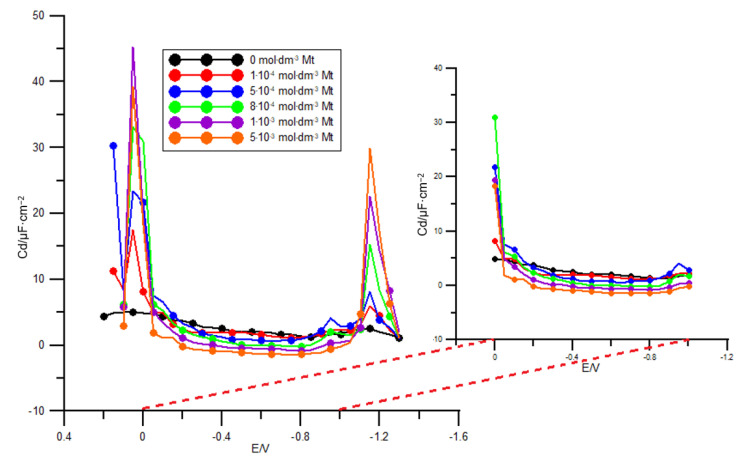
Differential capacity–potential curves of electric double layer at the R–AgLAFE/chlorate(VII) solution interface in the absence and in the presence of increasing concentration of methionine. The values of the methionine concentration are given in the plot.

**Figure 2:**
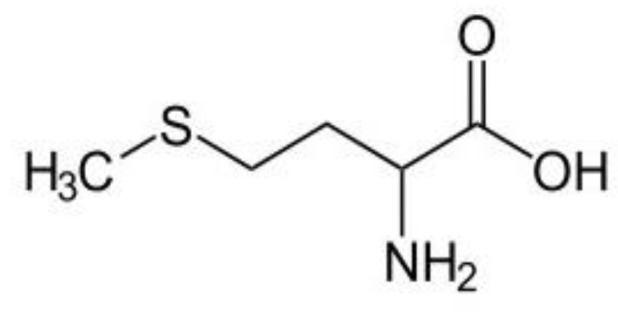
A scheme of the molecular structure of methionine.

Figure 1 presents the differential capacity curves of the double layer obtained by extrapolation to the zero frequency at the R–AgLAFE/chlorate(VII) solution interface in the presence of methionine. As can be inferred from Figure 1 a low concentration of methionine (1 × 10^−3^ mol dm^−3^) causes modification of the capacity curves, suggesting significant adsorption on the electrode surface, which is in accord with other findings [16,17]. Reduction in the differential capacity was observed with the increasing concentration of methionine in a wide range of potentials (from 0.2 to −1.0 V). Such evolution of the capacity curves indicates strong adsorption tendency of methionine [18]. In the region of high positive potentials (~0.0 V) the adsorption peak of methionine emerges (Figure 1), whereas in the region of negative potentials (~−1.1 V) desorption process takes place. The adsorption peak increases in proportion to the adsorbate concentration in the bulk of the supporting electrolyte. The region of potentials showing the adsorption and desorption peaks is probably an area of labile methionine adsorption [10,18]. The position of both adsorption and desorption peaks does not change with the methionine concentration, implying that adsorbed methionine molecules do not change their orientation on the electrode surface depending on the bulk concentration. Earlier studies have proved the specific methionine adsorption at the mercury/chlorate(VII) solution interface [18]. Moreover, it has been shown that methionine molecules are oriented with their negatively charged side towards the mercury electrode, which is the result of specific interactions between mercury and the sulphur atom of methionine (Figure 2) [18]. It seems plausible to assume similar arrangement of adsorbed methionine molecules on the R–AgLAFE.

### 2.2. Kinetics and Electrode Mechanism at the R–AgLAFE Electrode

The study of the electrode kinetics and mechanism of Bi(III) electroreduction at the novel R–AgLAFE [11] implies the catalytic effect of methionine following the “*cap*–*pair*” mechanism.

**Figure 3:**
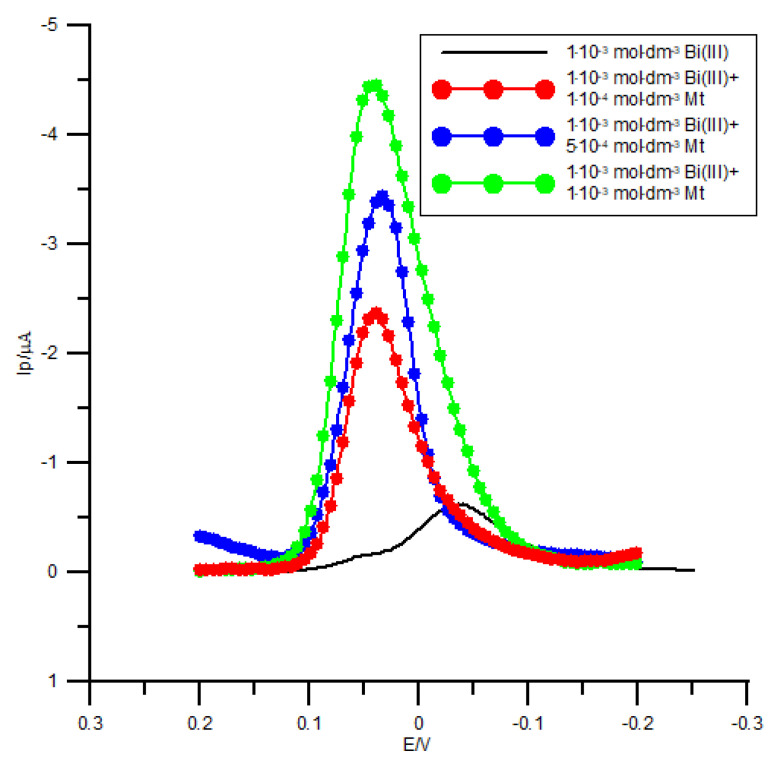
Net peaks of square-wave voltammetry (SWV) of 1 × 10^−3^ mol dm^−3^ Bi(III) electroreduction in 1 mol dm^−3^ chlorate(VII) medium in the absence (black curve) and in the presence of increasing methionine concentration. The concentration of methionine is given on the plot. The parameters of the potential modulation are: Step potential 2 mV, pulse amplitude 20 mV and frequency 120 Hz.

The net square-wave (SW) voltammograms in Figure 3 reveal clearly the effect of methionine on the electrode kinetics of Bi(III) electroreduction process. Methionine causes a remarkable enhancement of the net SW peak current and significant decrease of the half-peak width.

**Figure 4:**
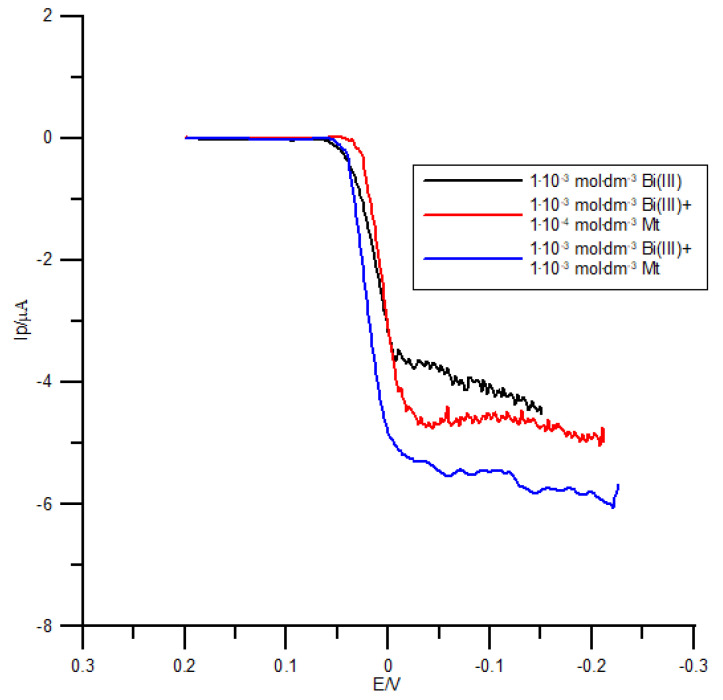
Direct current (DC) voltammograms of 1 × 10^−3^ mol dm^−3^ Bi(III) electroreduction in 1 mol dm^−3^ chlorate(VII) solution in the presence of methionine. The concentration of methionine is given in the plot. The step potential is 2 mV.

In addition, as can be inferred from Figure 4, the presence of methionine in the supporting electrolyte affects the slope of the linearly raising part of the direct current (DC) voltammograms for the Bi(III) electroreduction. Such voltammetric behaviour (Figure 3 and Figure 4) implies an increase in the electrochemical reversibility of Bi(III) electroreduction process at the R–AgLAFE [19,20].

**Figure 5:**
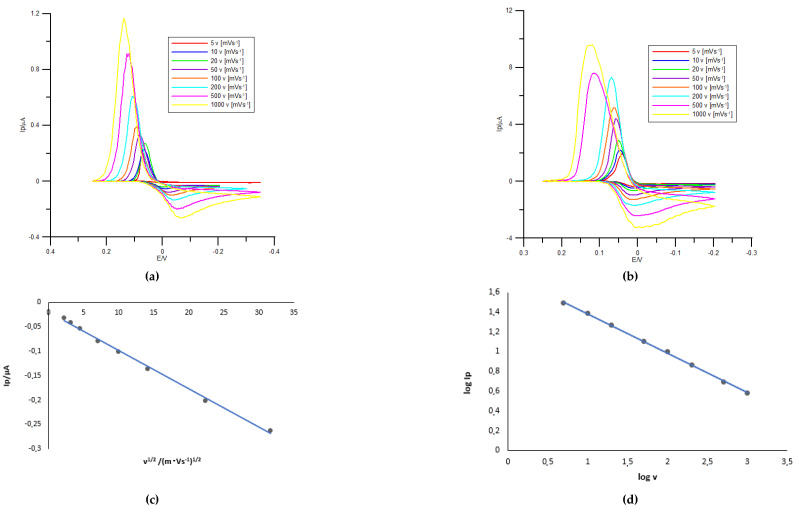

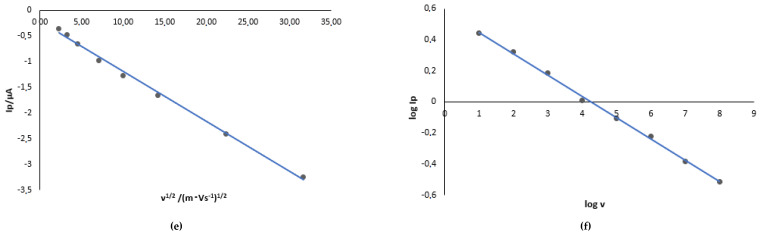
(**a**) Cyclic voltammograms of 1 × 10^−3^ mol dm^−3^ Bi(III) at different scan rates recorded in 1 mol dm^−3^ chlorate(VII) solution in the absence of the methionine. The scan rate (*v*) values are given on the plots. (**b**) Cyclic voltammograms of 1 × 10^−3^ mol dm^−3^ Bi(III) at different scan rates recorded in 1 mol dm^−3^ chlorate(VII) solution in the presence of 1 × 10^−3^ mol dm^−3^ methionine. The scan rate (*v*) values are given on the plots. (**c**) The dependence of the cathodic peak current for the reduction of Bi(III) (*I*_p_) on the square root of the scan rate (*v*^1/2^). Each point is an average of three measurements. (**d**) The log–log dependence of the reduction peak current and the scan rate over the interval from 5 to 1000 mV s^−1^ (in the absence of the methionine). Each point is an average of three measurements. (**e**) The dependence of the cathodic peak current for the reduction of Bi(III) + 1 × 10^−4^ mol dm^−3^ methionine (*I*_p_) on the square root of the scan rate (*v*^1/2^). Each point is an average of three measurements. (**f**) The log-log dependence of the reduction peak current and the scan rate over the interval from 5 to 1000 mV s^−1^ (in the presence of 1 × 10^−3^ mol dm^−3^ methionine). Each point is an average of three measurements.

As can be seen in Figure 5a, both oxidation and reduction peaks are well defined. Furthermore, when the scan rate was increased, the oxidation peak potential shifted toward more positive potential values while the reduction peak shifted toward more negative values. When methionine is present in the base electrolyte solution (Figure 5b), the peak potential changes are much smaller, indicating intermediate steps in the electrode process [21].

Cathodic peak of Bi(III) reduction in the absence (Figure 5a) and the presence of methionine (Figure 5b) varies linearly with the square root of the scan rate suggesting a diffusion-controlled process. In addition, the relationship between the logarithm of the peak current (log *I*_p_) and the logarithm of the scan rate (log *v*) in the absence of methionine is also linear (Figure 5d) with a slope near to 0.5 indicating a diffusion-controlled process. Similar relationships were observed in the system with methionine (Figure 5e,f).

**Figure 6:**
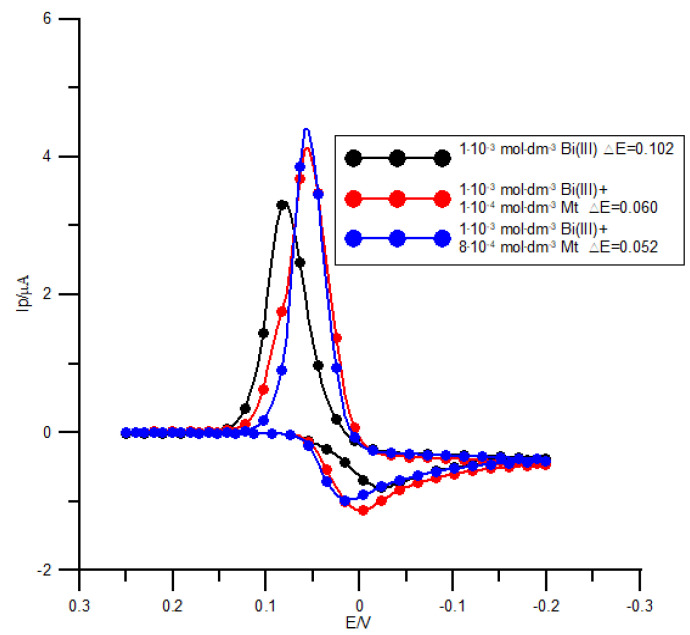
Cyclic voltammograms of 1 × 10^−3^ mol dm^−3^ Bi(III) electroreduction in 1 mol dm^−3^ chlorate(VII) solution in the presence of methionine. The concentration of methionine, together with the value of the corresponding peak potential separation, is given in the plot. The scan rate was 50 mV s^−1^.

The catalytic activity of methionine can be further confirmed by studying the electrode reaction with CV, which is mainly manifested by a decrease in the peak potential separation (Δ*E*) (Figure 6). However, the overall analysis of the peak potential separation as a function of the scan rate (Table 1) implies involvement of a chemical step in the electroreduction mechanism of Bi(III) in the presence of methionine [6,22].

**Figure 7:**
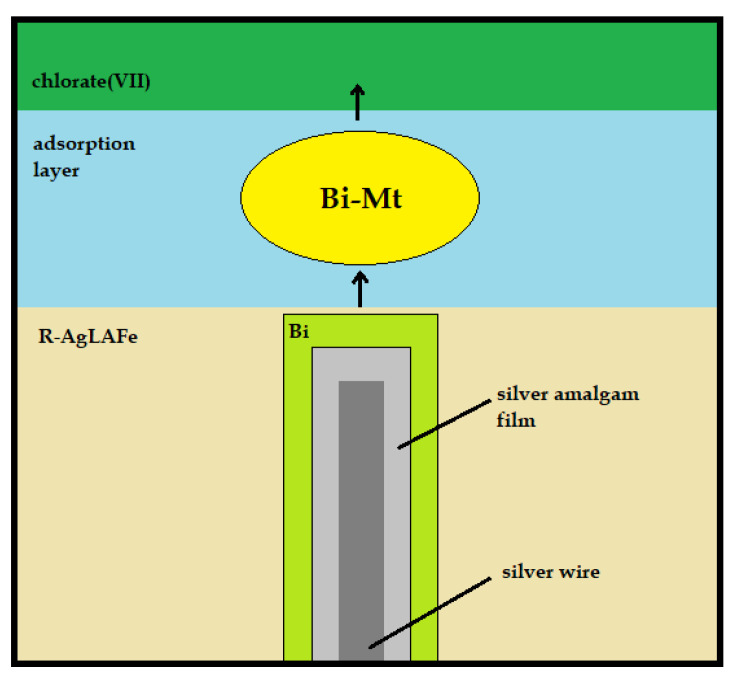
The reaction path obtained for Bi(III) ion electroreduction in the presence of methionine.

The previous studies using mercury electrode [22,23] indicated an essential role of the active Bi-Mt complex mediating the electron transfer process [23]. The adsorption studies suggest specific adsorption of methionine at the R–AgLAFE/chlorate(VII) interface; thus, formation of such complex is plausible at the amalgam electrode as well. Moreover, the value of the formal potential *E*^0^*_f_* of the Bi(III) electroreduction in the presence of methionine remains constant regardless of the particular methionine concentration (*E*^0^*_f,_*_Bi(III)_ = 0.075 V; *E*^0^*_f,_*_Bi(III)+1×10_^−4^_Mt_ = 0.078 V; *E*^0^*_f,_*_Bi(III)+5×10_^−4^_Mt_ = 0.077 V; *E*^0^*_f,_*_Bi(III)+8×10_^−4^_Mt_ = 0.078 V; *E*^0^*_f_*_Bi(III)+1×10_^−3^_Mt_ = 0.079 V; *E*^0^*_f,_*_Bi(III)+5×10_^−3^_Mt_ = 0.078 V), which supports the above assumption. The active Bi(III)-Mt complexes are most probably localized inside the adsorption layer (Figure 7) [22,23,24].

**Figure 8:**
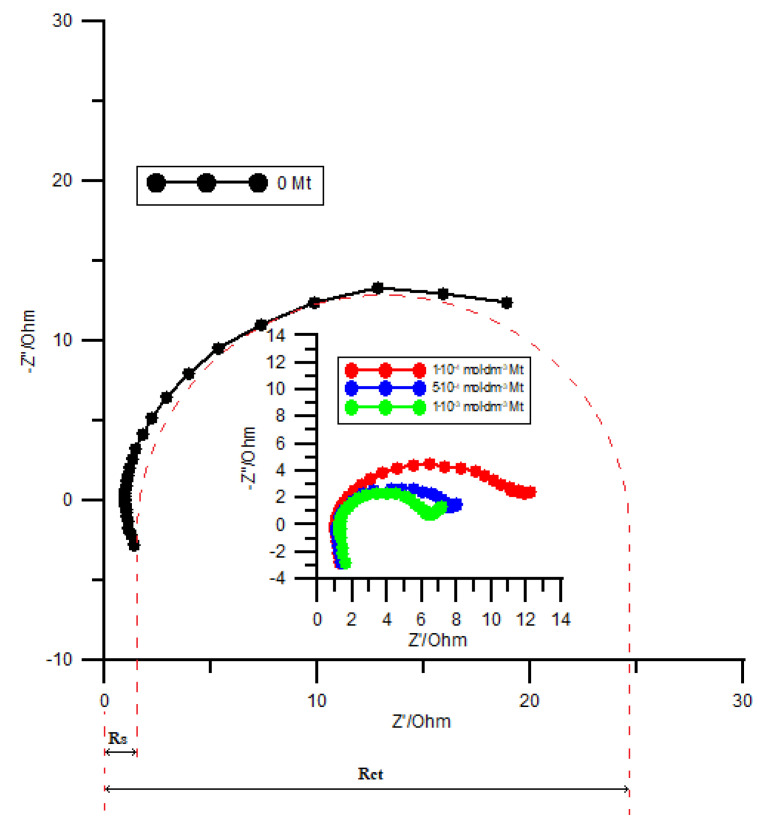
Impedance diagrams measured at *E_f_*^0^ for the electroreduction of 1 × 10^−3^ mol dm^−3^ Bi(III) in 1 mol dm^−3^ chlorate(VII) solution in the presence of methionine. The values for the concentration of methionine are given in the plot.

As follows from the data collected by electrochemical impedance spectroscopy conducted at the formal potential values (Figure 8), the addition of the methionine in the supporting electrolyte results in a decrease of the resistance (*R*_ct_) due to the charge transfer. The charge transfer resistance is derived from the overpotential *η*; hence, a decrease in *R*_ct_ is associated with a decrease in the overpotential for the reaction under study. The lower the charge transfer resistance (i.e., according to Figure 8—higher methionine concentration), the lower the overpotential [25]. The reason for this dependence is likely to be the mediation of the charge transfer across the interface by methionine molecules adsorbed on the electrode surface or by formation of active methionine complexes with bismuth ions, as previously mentioned. This is another support for the catalytic activity of methionine on the electroreduction process of Bi(III) ions in a chlorate(VII) solution.

**Figure 9:**
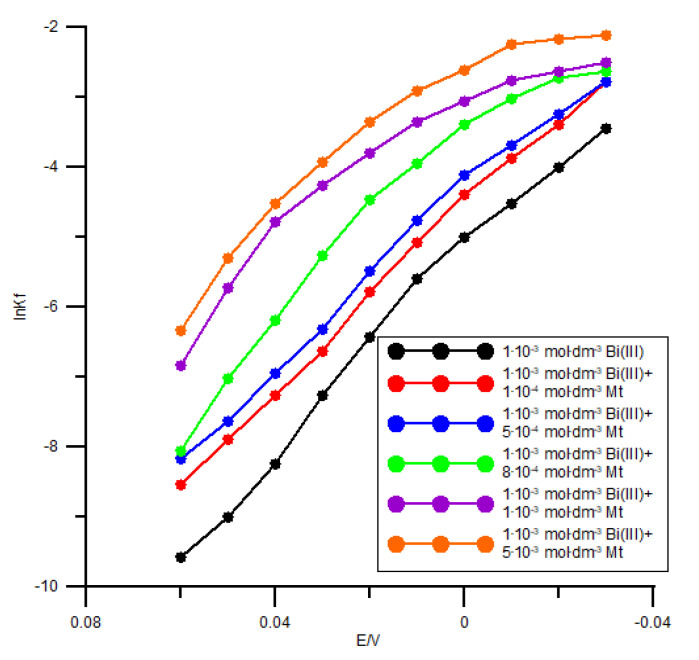
The dependence of the rate constants *k_f_* on the electrode potential for the electroreduction of 1 × 10^−3^ mol dm^−3^ Bi(III) in 1 mol dm^−3^ chlorate(VII) solution in the presence of methionine. The concentration of methionine is given on the plot.

The actual rate constant *k_f_* (*k_f_* values were computed from *R_ct_* [Equation (5)]) for the Bi(III) electroreduction in the presence of methionine, plotted as a function of the potential, are not linear (Figure 9), in accordance with the literature data [26].

These data imply a multistep and complex character of the electrode process [7,27], as suggested by Lovrić et al. [7]. The electrode mechanisms are a combination of a reaction step of pure chemical nature, which is independent of the electrode potential (e.g., a partial dehydration of the Bi(III) ion), with the potentially dependent steps involving electron transfer. The data of Figure 9 additionally indicate an effect of methionine on the transfer of the first electron (higher slope of the curves at more negative potentials), indicating formation of the Bi-Mt complex before the transfer of the first electron [2]. This stage is the slowest and thus determines the rate of the whole process. It should, however, be emphasized that the complex species are involved in the further electron exchange processes at the electrode interface. The study based on the Marcus electron-transfer theory [28] assumes different compositions of these complexes. Similar assumption has been made in the studies using the mercury electrode [3,18,22,24].

The obtained values of the kinetic parameter, i.e., the catodic electron transfer coefficient *α* (*α*_Bi(III)_ = 0.28; *α*_Bi(III)+1×10_^−4^_Mt_ = 0.33; *α*_Bi(III)+5×10_^−4^_Mt_ = 0.38; *α*_Bi(III)+8×10_^−4^_Mt_ = 0.40; *α*_Bi(III)+1×10_^−3^_Mt_ = 0.48; *α*_Bi(III)+5×10_^−3^_Mt_ = 0.52) confirm the catalytic effect of methionine on the electroreduction of Bi(III) ions in the chlorate(VII) solutions [6]. The same can be concluded from the values of the standard rate constant *k_s_* (Table 2). It should be pointed out that the rate constants determined from the CV and EIS measurements are consistent.

## 3. Experimental

### 3.1. Chemicals

All reagents, NaClO_4_, HClO_4_, Bi(NO_3_)_3_∙5H_2_O and methionine (Fluka), were of analytical reagent grade. Water was purified with MilliporeMilli-Q system (Merck KGaA, Darmstadt, Germany). The supporting electrolyte was 0.5 mol dm^−3^ NaClO_4_ + 0.5 mol dm^−3^ HClO_4_. The concentration of Bi(III) ions in the solutions was 1 × 10^−3^ mol dm^−3^. Due to poor solubility of Bi(NO_3_)_3_ in chlorate(VII) solution the solutions were treated by ultrasound. The concentration of methionine was set to 1 × 10^−^^4^ and 5 × 10^−3^ mol dm^−^^3^.

### 3.2. Apparatus

**Figure 10:**
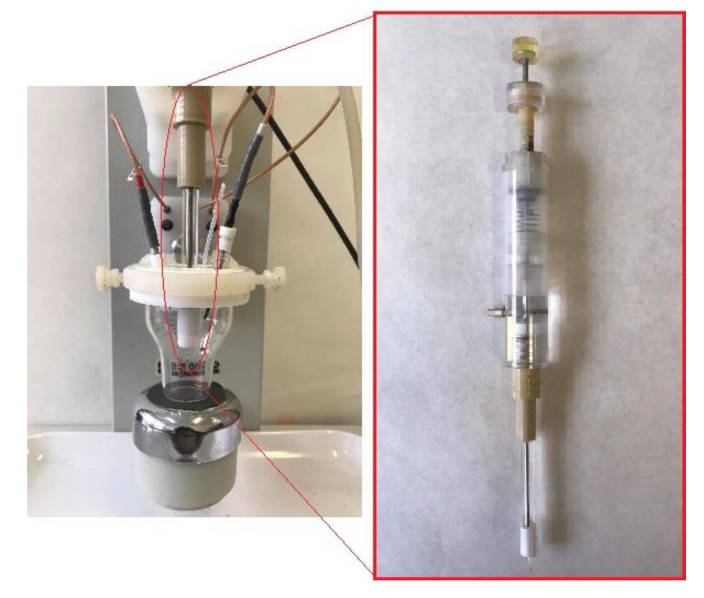
Three-electrode voltammetric cell with the centrally fixed working electrode (R–AgLAFE).

All electrochemical measurements were performed with Autolab Fra 2/ GPES (Version 4.9) frequency response analyser (Eco Chemie, Utrecht, Netherlands). A three-electrode system (Figure 10) was composed of Ag/AgCl/3M KCl electrode as a reference, a platinum wire as an auxiliary electrode and cyclically renewable liquid silver amalgam film electrode (R–AgLAFE), which was renewed prior to each measurement with a surface area of 17.25 mm^2^ [10], as a working electrode.

Additionally, the working electrode surface morphology was examined using an optical microscope Nikon Eclipse MA200 with the lens “Nikon Lu Plan Fluor 10x/0.30A” and the polarization filter “MA2-PA”.

**Figure 11:**
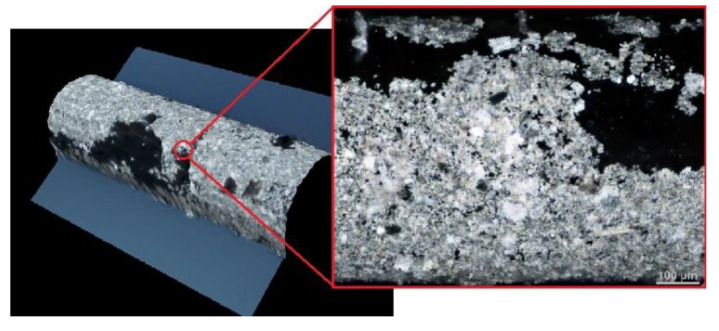
R–AgLAFE surface seen as an image from a Nikon Eclipse MA200 optical microscope with a “Nikon Lu Plan Fluor 10x/0.30A” objective.

Figure 11 shows that the liquid supersaturated (1% (*w*/*w*)) silver amalgam forms a thin film on the surface of silver base (wire) without destruction of its texture [10]. The slight discontinuity of the film is caused by the small mechanical strength during electrode preparation. However, it does not affect the reproducibility of the voltammetric curves. All electrochemical measurements were done at 298 K.

### 3.3. Measurement Procedures

#### 3.3.1. Adsorption Procedure

##### Experimental Operating Conditions

The differential capacity of the double layer (C_d_) at the R–AgLAFE/supporting electrolyte interface was measured by means of the impedance spectroscopy. In order to achieve the capacity dispersion for the whole polarisation range this was performed at different frequencies between 200 and 1000 Hz. To get the appropriate equilibrium values of differential capacity, a linear dependence of capacity on the square element from the frequency was extrapolated to the zero frequency. The procedure is based on the theory that the impedance of the double layer is equivalent to a series of capacity-resistance combinations and the rate of adsorption is diffusion controlled [29].

#### 3.3.2. Kinetic Procedure

##### Experimental Operating Conditions

In the DC voltammetry, SWV and CV voltammetry, the optimal experiment operating conditions were as follows: Step potential of 2 mV for DC; pulse amplitude 20 mV, frequency 120 Hz and step potential 2 mV for SWV; and scan rate 5–1000 mV s^−1^ with a step potential of 5 mV for CV. The electrochemical impedance spectroscopy data were collected at 36 frequencies in the range from 15 to 50,000 within the faradaic potential region with 10 mV intervals.

##### Elaboration of Experimental Data

The formal potentials ($E_{f}{}^{0}$) for electrode processes were determined using Randles [29] modified method from the equation:

(1) $$E_{f}{}^{0}=\frac{1}{2}\left[E_{a/4}+E_{c/4}+\frac{\left(E_{a/4}+E_{c/4}\right)-\left(E_{3a/4}+E_{3c/4}\right)}{g-1}\right]$$

where:

$$g=\frac{\left(E_{3a/4}-E_{3c/4}\right)}{\left(E_{a/4}-E_{c/4}\right)}$$

$E_{a/4}\;$ or $E_{3a/4}$—the potentials of accordingly one fourth or three fourths of the anodic peak height; $E_{c/4}$ or $E_{3c/4}$—the potentials of accordingly one fourth or three fourths of the cathodic peak height of cyclic voltammograms.

The catodic electron transfer coefficient *α* was based on the following equation [29]:

(2) $$\alpha n_{\alpha }=\frac{0.048}{E_{pk/4}-E_{3pk/4}}$$

The standard rate constants were determined considering criteria for the electrochemical reversibility of the studied process as follows [29]:

For an irreversible electrode process (i.e., electroreduction of 1 × 10^−3^ mol dm^−3^ Bi(III)) in a chlorate(VII) medium:

(3) $$E_{pk}=E_{f}^{0}-\frac{RT}{\alpha n_{\alpha }F}\left[0.78-lnk_{s}+ln\sqrt{D_{ox}b}\right]$$

For the quasireversible process (i.e., the electroreduction of 1 × 10^−3^ mol dm^−3^ Bi(III)) in the chlorate(VII) medium in the presence of methionine:

(4) $$\Psi ={\left(\frac{D_{ox}}{D_{red}}\right)}^{\alpha /2}\frac{k_{s}{\left(RT\right)}^{1/2}}{{\left(\pi nF\nu D_{ox}\right)}^{1/2}}$$

The values of the apparent rate constants ($k_{f}$) of Bi(III) ion electroreduction in the studied solutions as a function of potential were calculated from impedance measurements.

$k_{f}$ values were computed from $R_{ct}$ values as a function of DC potential [23].

(5) $$R_{ct}=\frac{RT}{n^{2}F^{2}c_{0}k_{f}S}\cdot \frac{a_{0}/k_{f}+1+r_{s}exp(b)}{\alpha a_{0}/k_{f}+r_{s}exp(b)}$$

The details of the determination of the above parameters are described elsewhere [23].

## 4. Conclusions

Based on the results obtained at the R–AgLAFE with the automatically renewable working film the electroreduction of Bi(III) ion in the presence of methionine proceeds according to the “*cap***–***pair*” mechanism. The rate determining step is the dehydration of the bismuth hydro-complex ${\left[\mathrm{Bi}{\left(H_{2}O\right)}_{9}\right]}^{3+}$; thus, the catalytic effect of methionine is assumed to be related to the replacement of coordinated water molecules from the inner hydration shell of the Bi(III) ion. Introducing a foreign ligand (such as methionine) into the coordination sphere of the hydro-complex increases the rate of displacement of the remaining water molecules. Hence, labialization of the hydration sphere probably plays a key role in many ligand catalyzed reductions of metal ions. This reaction occurs preferentially on the electrode surface because of the significant local methionine concentration existing as a result of methionine adsorption, forming favorable conditions for Bi–methionine complexes formation. The active complexes participate in the transfer of consecutive electrons.

The following mechanism of the catalytic effect of the methionine on Bi(III) ions electroreduction in a chlorate(VII) solution can be assumed:

- partial dehydration of Bi(III) ions and formation of active complex (I)

$$Bi{\left(H_{2}O\right)}_{9}^{3+}\;+\;x{\left(Mt\right)}_{ads}\;\to \;Bi{\left(H_{2}O\right)}_{\left(9-a\right)}^{3+}{\left(Mt\;\right)}_{x}\;+\;aH_{2}O$$

- first electron transfer

$$Bi{\left(H_{2}O\right)}_{\left(9-a\right)}^{3+}{\left(Mt\;\right)}_{x}\;+\;\bar{e}\;\to \;Bi{\left(H_{2}O\right)}_{\left(9-a\right)}^{2+}{\left(Mt\;\right)}_{x}$$

- further dehydration and formation of active complex (II)

$$Bi{\left(H_{2}O\right)}_{\left(9-a\right)}^{2+}{\left(Mt\;\right)}_{x}\;\pm \;y{\left(Mt\;\right)}_{ads}\;\to Bi{\left(H_{2}O\right)}_{\left(9-a-b\right)}^{2+}{\left(Mt\right)}_{x\;\pm \;y}\;+\;bH_{2}O$$

- second electron transfer

$$Bi{\left(H_{2}O\right)}_{\left(9-a-b\right)}^{2+}{\left(Mt\;\right)}_{x\;\pm \;y}\;+\;\bar{e}\;\to \;Bi{\left(H_{2}O\right)}_{\left(9-a-b\right)}^{+}{\left(Mt\;\right)}_{x\;\pm \;y}$$

- dehydration of Bi(III) ions and formation of active complex (III)

$$Bi{\left(H_{2}O\right)}_{\left(9-a-b-c\right)}^{+}{\left(Mt\;\right)}_{x\;\pm \;y}\;\pm \;z{\left(Mt\;\right)}_{ads}\;\to \;Bi^{+}{\left(Mt\;\right)}_{x\;\pm \;y\pm z}\;+\;\left(9-a-b-c\right)H_{2}O$$

- third electron transfer and amalgam formation

$$i^{+}{\left(Mt\;\right)}_{x\;\pm \;y\pm z}\;\bar{e}\;\to \;Bi\left(Hg\right)\;+\;\left(x\pm y\pm z\right){\left(Mt\;\right)}_{ads}$$

The obtained results with the R–AgLAFE for the mechanism and kinetics of an electrode reaction following the “*cap***–***pair*” pathway suggest that the electrode is an attractive alternative to conventional HMDE. The practical aspect of this research is connected with the possibility of directing and finding new ways for determination of Bi(III) ions as well as methionine.

## Figures and Tables

**Table 1:** The peak potential separation Δ*E* of cyclic voltammograms for the electroreduction of 1 × 10^−3^ mol dm^−3^ Bi(III) in 1 mol dm^−3^ chlorate(VII) solution in the presence of methionine for different scan rates *v*.

10^3^ C_Bi(III)_ + 10^4^ C_Mt_/mol dm^−3^	ΔE/V							
	v/mV s^−1^							
	5	10	20	50	100	200	500	1000
0.00	0.098	0.097	0.100	0.102	0.110	0.126	0.148	0.171
1.00	0.059	0.058	0.060	0.065	0.071	0.078	0.103	0.147
5.00	0.056	0.056	0.057	0.058	0.068	0.070	0.082	0.125
8.00	0.055	0.054	0.053	0.052	0.060	0.067	0.096	0.113
10.0	0.048	0.049	0.050	0.051	0.058	0.061	0.090	0.110
15.0	0.040	0.041	0.044	0.045	0.052	0.055	0.082	0.093

**Table 2:** The values of standard rate constants *k_s_* of 1 × 10^−3^ mol dm^−3^ Bi(III) electroreduction in 1 mol dm^−3^ chlorate(VII) and in the presence of methionine estimated with CV and EIS.

10^3^ C_Bi(III)_ + 10^4^ C_Mt_/mol dm^−3^	10^4^*k_s_*/cm s^−1^	
	CV	EIS
0.00	0.35	0.40
1.00	4.23	4.30
5.00	6.74	6.30
8.00	8.19	8.60
10.0	9.46	9.20
50.0	10.3	11.6

## Data Availability

Data are available from the authors by request.
